# The Host Cytoskeleton Functions as a Pleiotropic Scaffold: Orchestrating Regulation of the Viral Life Cycle and Mediating Host Antiviral Innate Immune Responses

**DOI:** 10.3390/v15061354

**Published:** 2023-06-12

**Authors:** Meilin Li, Dingkun Peng, Hongwei Cao, Xiaoke Yang, Su Li, Hua-Ji Qiu, Lian-Feng Li

**Affiliations:** State Key Laboratory for Animal Disease Control and Prevention, Harbin Veterinary Research Institute, Chinese Academy of Agricultural Sciences, Harbin 150069, China

**Keywords:** host cytoskeleton, viral replication cycle, host–virus interactions, orchestrated crosstalk

## Abstract

Viruses are obligate intracellular parasites that critically depend on their hosts to initiate infection, complete replication cycles, and generate new progeny virions. To achieve these goals, viruses have evolved numerous elegant strategies to subvert and utilize different cellular machinery. The cytoskeleton is often one of the first components to be hijacked as it provides a convenient transport system for viruses to enter the cell and reach the site of replication. The cytoskeleton is an intricate network involved in controlling the cell shape, cargo transport, signal transduction, and cell division. The host cytoskeleton has complex interactions with viruses during the viral life cycle, as well as cell-to-cell transmission once the life cycle is completed. Additionally, the host also develops unique, cytoskeleton-mediated antiviral innate immune responses. These processes are also involved in pathological damages, although the comprehensive mechanisms remain elusive. In this review, we briefly summarize the functions of some prominent viruses in inducing or hijacking cytoskeletal structures and the related antiviral responses in order to provide new insights into the crosstalk between the cytoskeleton and viruses, which may contribute to the design of novel antivirals targeting the cytoskeleton.

## 1. Introduction

**Figure 1:**
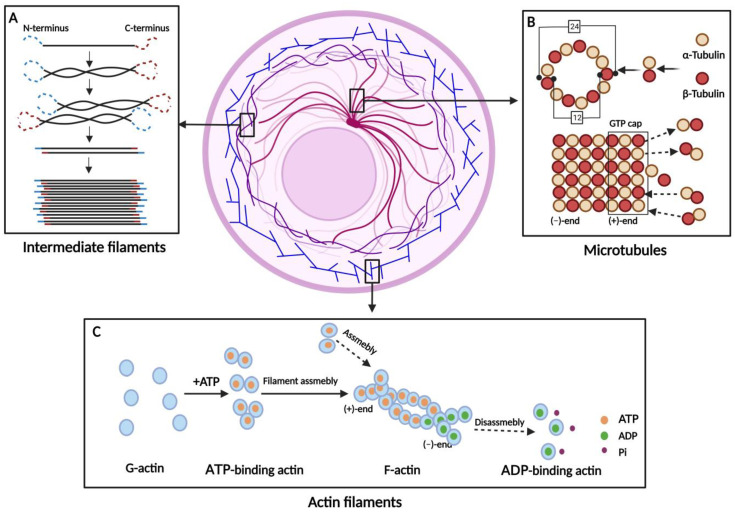
Schematic diagram of the distribution and structure of the cytoskeleton in the cell. (**A**) Intermediate filaments are formed by the spiral aggregation of monomers into dimers, followed by the aggregation of two dimers into a tetramer; finally, eight tetramers assemble to create a unit-length filament. (**B**) Microtubules (MTs) are composed of α- and β-heterodimers, which assemble into a hollow tube structure. The elongation of MTs occurs via the addition of heterodimers, resulting in the formation of a GTP cap at the positive end of the MTs. (**C**) Actin filaments are formed via a multistep process that begins with the binding of G-actin monomers to ATP. Following this step, G-actin monomers associate to form unstable dimers or trimers, which then elongate the filament. At the positive end, ATP-binding actin will be assembled and the ATP will gradually be hydrolyzed into ADP and Pi, once a G-actin is added to the filament. The minus end of an F-actin filament often contains the actin molecules in an ADP-binding form.

As an essential part of maintaining the normal function of cells, the cytoskeleton plays important roles in the activities of life, including endocytosis, cell division, intracellular transport, motility, force transmission, reactions to external forces, adhesion and preservation, and cell shape adaptation. The cytoskeleton is mainly composed of three types of cytoskeletal polymers, including actin filaments (AFs), microtubules (MTs), and intermediate filaments (IFs) [1]. AFs, MTs, and IFs constitute a complex network involved in the functions of eukaryotic cells, providing cells with the ability to perform multiple functions uniformly [2]. These proteins assemble into different structures to play broad roles [3], and form highly structured and dynamic networks. An intricate network of components is capable of swift adaptation in response to both external and internal stimuli, enabling precise regulation within minutes [1]. These three cytoskeletal proteins have different functions, but they are mutually regulated and work together to complete vital movement [4]. Information related to the cytoskeleton is summarized (Table 1 and Figure 1).

When the normal physiological activities of cells are disturbed, the cytoskeleton also undergoes remarkable changes accordingly, and abnormal conditions usually occur when the cells themselves propagate out of control or are disturbed by exogenous substances [5]. Cytoskeletal alterations contribute to the spread and migration of cancer cells [6]. Changes in cytoskeletal proteins in passively infected cells can also affect the infection process of microorganisms, such as viruses, bacteria, and parasites [5].

The cytoskeleton plays an active role in the viral life cycle. The process involves the virus invading the cell, traveling to the replication site, localizing the viral components to the proper assembly site after replication, and transporting them to the viral budding site. Since cortical actin affects the deformation of the cell membrane [7], virus entry into the cell must be regulated by actin [8]. Considering the capacity of MTs to facilitate intracellular transportation, their potential involvement in the viral life cycle is worth exploring. Many viral replicative mechanisms have been studied, and it has been found that microtubules can be used by viruses to transfer materials [9]. Upon viral infection, vimentin is crucial for stress response and signal transduction in cells [10]. This process assists the virus in propagating once it enters the cell [11]. In addition, viruses can use the cytoskeleton to spread from one cell to another and form a connecting channel between the two cells, which plays a significant role in their pathogenesis [12]. Recent research has confirmed that the cytoskeleton regulates the signaling pathway of IFN [13,14,15].

Current data show that the functions of the cytoskeleton are diverse. On one hand, the virus hijacks the cytoskeleton to complete its life cycle; on the other hand, the cytoskeleton assists cells to complete the process of innate immunity. This article reviews the functions of the cytoskeleton in viral colonization and propagation, including the process of virus invasion and host antiviral response.

**Table 1:** Structure and functions of the cytoskeleton.

Cytoskeleton Types	Main Members	Polymer Formation	Functions	References
Actin filaments (AFs)	*β*-Actin *γ*-Actin	G-actin forms an unstable dimer or trimer, and then the filaments are elongated by the addition of monomers.	Muscle contraction/ Maintenance of cell surface shape/ Deformable movement/ Cytokinesis	[1,16,17,18]
Microtubules (MTs)	*α*-Tubulin *β*-Tubulin	*α*- and *β*-Tubulin form a heterodimer, which is continuously extended. Thirteen extended tubulin protofilaments form a hollow tube.	Maintaining cell shape/ Transport of substances/ Assistant in mitosis	[1,19,20,21,22,23,24]
Intermediate filaments (IFs)	Acidic Keratins Basic Keratins Vimentin Lamins	IFs arise from the monomers spiraling around each other to form dimers. Two dimers aggregate to a tetramer and eight tetramers to a unit-length filament.	Maintaining cell morphology/ Signal transduction/ Involved in cellular stress	[1,25,26,27,28,29]

## 2. Physiological Functions of the Cytoskeleton on Normal Conditions

AFs are the major structural components of cells, and actin is the most abundant protein in many eukaryotic cells [30]. Actin is a 42-kDa protein with 375 amino acids and is highly conserved across a variety of species. It has six isoforms and more than 60 proteins [31], and only *β*-actin and *γ*-actin are expressed in most mammalian cell types [32]. The monomeric form of actin, known as G-actin, is the most fundamental structure for actin to perform its biological activities. Through a double or triple helix, G-actin creates the dimer or trimer F-actin, which is 7 nm in diameter. G-actin and F-actin serve different purposes, and the formation between them changes continually, preserving a relative balance in the absence of a stimulus. Actin has an ATP-binding region at its center, which binds to ATP in order to aggregate. The positive end of F-actin continuously binds to G-actin with ATP, extending into filament. ATP at the negative end will hydrolyze into ADP and Pi, resulting in the depolymerization of F-actin [1]. The equilibrium between the two formations is destroyed when the cellular activities change. For example, AFs prefer to polymerize when they are required to maintain cell morphological stability, whereas they typically depolymerize when they are required for cell deformation and movement [33]. Actin-binding proteins (ABPs) regulate F-actin [34]. For example, the actin-related protein (Arp2/3) is an ABP that drives G-actin polymerization to form F-actin, which can be activated by GTP depletion [35].

Actin, regarded as the most dynamic among the three major cytoskeletal proteins, is capable of rapid and significant structural alterations within minutes, which crucially contribute to determining cellular morphology [1]. Actin is involved in many physiological processes including cell motility, division, differentiation, senescence, death cell motility, division, differentiation, senescence, and death [36]. In all eukaryotic cells, actin regulates most cellular functions, including intercellular adhesion, cell motility, and cell division. The actin cytoskeleton is located in the cytoplasmic side of the plasma membrane and consists of a filamentous F-actin network that interfaces with the plasma membrane via surface receptors. Cortical actin is involved in all events related to the expression and presentation of membrane and cell surface molecules, the formation and movement of endocytic and phagocytic vesicles, viral entry, exocytosis, and viral export [16]. Actin regulates DNA repair, chromatin remodeling condensation, and gene transcription in the nucleus [17]. Moreover, nuclear actin has been identified as a crucial component of chromatin remodeling complexes that regulate gene expression. It interacts with all three RNA polymerases and plays a critical role in transcription initiation and elongation [37].

Long tubular organelles, known as MTs, are essential in eukaryotic cells and play a significant role in the cell cycle. These genes are highly conserved in many species. *α*-Tubulin is composed of 450 amino acid residues and *β*-tubulin is composed of 455 amino acid residues, which have a molecular weight of approximately 55 kDa. It has an average outer diameter of 24 nm and an inner diameter of 12 nm [1], and its structure is composed of *α*-tubulin and *β*-tubulin heterodimers assembled into a hollow polymer [19]. *α*-Tubulin and *β*-tubulin are sequentially arranged to form a single fiber, and 13 such fibers are arranged to form a hollow tubular structure [1]. The slower ends of polymerization and dissociation are the negative end of MTs, and this portion of *α*-tubulin is exposed. Faster polymerization and dissociation occur at the positive end of the microtubule exposed to *β*-tubulin [19]. Since a GTP cap exists at the microtubule positive ends, polymerization and depolymerization can be quickly completed in the cell, and are responsible for microtubule mass formation and dynamic interactions with different subcellular structures [20]. It also points to the positive end of the plasma membrane, which contributes to the intracellular trafficking of MT-bound vesicles. Owing to the growth and shortening of the positive ends, microtubule dynamics are generated [21]. MTs are altered by GTP hydrolysis for energy. GTP can be bound to *α*-tubulin, whereas GTPase is present on *β*-tubulin. Microtubule-binding proteins (MBPs) can directly or indirectly bind to MTs to regulate their dynamics, assembly, disassembly, and stability [22]. In contrast, the negative ends are hardly involved in depolymerization and polymerization, and determine the geometry of MTs network. Therefore, they are usually stably anchored at the microtubule nucleation sites [23]. The development of various forms of microtubule-organizing centers (MTOCs) results in a highly polymerized tubulin-containing structure, while *γ*-tubulin is highly polymerized [38]. In most cells, MTs are radially distributed around the cell from the center of MTOCs, with the positive pole pointing toward the cell membrane [1]. Microscopic motor proteins are divided into dynein and kinesin. Dynein transports intracellular material toward MTOCs, whereas kinesin transports intracellular materials toward the cell membrane. Intracellular vesicles can then be transported to different organelles through MTs, which is of great significance for efficient functioning [24]. The functional roles of MTs can be categorized into three distinct areas: cell motility, signal transduction regulation, and intracellular transportation [1].

IFs are fundamental building blocks of the cellular architecture and are generated from a vast array of proteins encoded by at least 70 genes [25]. The molecular weight of the protein is 52–58 kDa. Among these, vimentin is relatively conserved across various species, with a molecular weight ranging from 52 to 58 kDa. The structure of IFs consists of an N-terminus, a central *α*-helical rod domain, and a C-terminus of varying lengths [26]. During the interaction, the N- and C-termini of the monomer remain unwound, whereas the middle segment forms a parallel dimer, subsequently forming an antiparallel tetramer. Eight of these tetramers form a single filament with a compressed diameter of 10 nm [39]. IFs are classified into five categories based on their structural composition and sequence homology. Types I and II are acidic and basic keratins that form a heteropolymeric structure comprising 54 distinct subtypes of type III IFs, including vimentin [27], whereas type IV IFs are primarily expressed in the nervous system and contain three neurofilament heteropolymers (NF-L/M/H): synemin, internexin, and nestin. Type V IFs are nuclear filaments called lamins, and consist of A/C, B1, and B2 lamins [1]. Vimentin interacts with a range of proteins and performs crucial biological functions in IFs networks. Lysosomal and aggregate localization, cell migration, and various organelles and cellular components can be fixed within a specific range using the vimentin framework [28]. Vimentin, which is critical for multiple cellular functions, can sense and respond to cellular stress, including oxidative stress [29]. Vimentin can bind to the NF-*κ*B sites, thereby changing the immune response [15], which is also critical for intracellular signal transduction.

## 3. Pathological Roles of the Cytoskeleton on Abnormal Conditions

### 3.1. Neoplasm and Cancer

The cytoskeleton is essential for cancer progression, and contributes to the metastasis and spread of tumor cells by maintaining the cell shape, movement, and other functions. Actin remodeling can promote tumor invasive growth and tumor cell proliferation in the skeletal muscle [40]. In addition, Rho small GTPases belong to the Ras superfamily of GTPases, which regulate a wide array of cellular processes related to their key roles controlling the cytoskeleton. Rho-GTPase is an important player in key signaling pathways that regulate cell migration, such as cytoskeletal dynamics, the assembly and disassembly of cell–cell connections, directional sensing, and integrin–matrix adhesion [41]. Rho-GTPases, their modulators, and effectors are involved in several aspects of cancer progression [42]. As an illustration, epithelial mesenchymal transition (EMT) is the process through which epithelial cells transform into mesenchymal cells. Once epithelial cells undergo EMT, they reorganize the cytoskeleton and change the signalling programs that define the cell shape and reprogram gene expression, and individual cells become more aggressive as a result [43]. EMTs are also influenced by changes in the cytoskeleton, such as altered intermediate filament composition caused by the inhibition of cytokeratin and the activation of vimentin [44]. This enables cell motility in response to changes in the structure of IFs, possibly as a result of the interaction of vimentin with motor proteins [45].

### 3.2. Passive Infection with Bacteria, Viruses, or Parasites

Viruses, bacteria, parasites, and other microorganisms infect cells, which then produce corresponding countermeasures. The most significant change that occurs as a result of this conversion is the recombination of actin [46]. Bacterial infections and inflammation can disrupt the epithelial barrier, and host cell cytoskeletal changes in the host cell can directly mediate bacterial invasion into the intracellular environment [47]. In the intracellular niche, some bacteria then utilize the host cytoskeletal network to spread from cell to cell [48]. The binding of the *Listeria monocytogenes* surface proteins (InlA) to cell receptors promotes two posttranslational modifications of E-cadherin, primarily comprising host kinase Src phosphorylation followed by ubiquitination by the E3 ubiquitin ligase Hakai. This actin undergoes polymerization, which is a key molecular event required for virus entry into the cell [49]. The dissemination process of the intracellular pathogen *Shigella* primarily relies on actin assembly at the bacterial pole, propelling the pathogen throughout the infected cells [50]. Vimentin plays a role in facilitating bacterial transport, leading to subsequent immune inflammatory responses [51]. Viral infection alters normal cytoskeletal functions to optimize viral replication and virions production. Rabies virus (RABV) causes dendrite damage and actin depolymerization due to a reduction in actin fragments in nerve cells [52]. It also regulates the gene expression of cytoskeleton-related proteins and disrupts biological pathways that require cytoskeletal proteins [53]. Plasmodium and other intracellular parasites can use host factors such as hemoglobin S and C to modify and reshape the actin cytoskeleton network, thus changing the cargo transport mode of the organism and protecting patients from infection (Table 2) [54].

### 3.3. Pathological Process

The links between viral infection, cell morphology and changes in the actin cytoskeleton were determined using the description of the transformation [56]. The syncytium is formed by the fusion of cells of one or more species, which requires the rupture and reconnection of adjacent cell membranes. This entire process involves the support of the actin cytoskeleton beneath the cell membrane [57]. The pathogen responsible for COVID-19, severe acute respiratory syndrome coronavirus 2 (SARS-CoV-2), induces syncytia formation, which can increase the spread of the virus and facilitate the elimination of immune cells [58].

The disruption is also associated with neurodegenerative diseases. For example, infection with mouse hepatitis virus (MHV) induces tau phosphorylation through a mechanism dependent on glycogen synthase kinase-3*β*, which disrupts the stabilizing capacity of MTs, potentially leading to brain damage [59]. In Middle East respiratory syndrome coronavirus (MERS-CoV) and SARS-CoV-2 infections, troponin attaches to AFs and the level of troponin in the heart muscle of patients increases [60].

The influence of the cytoskeleton on viruses has been found to be significant. Here, what occurs when viruses hijack the cytoskeleton from various perspectives is discussed.

## 4. Multiple Engagements of the Cytoskeleton in Viral Life Cycle by Targeting Various Stages

**Figure 2:**
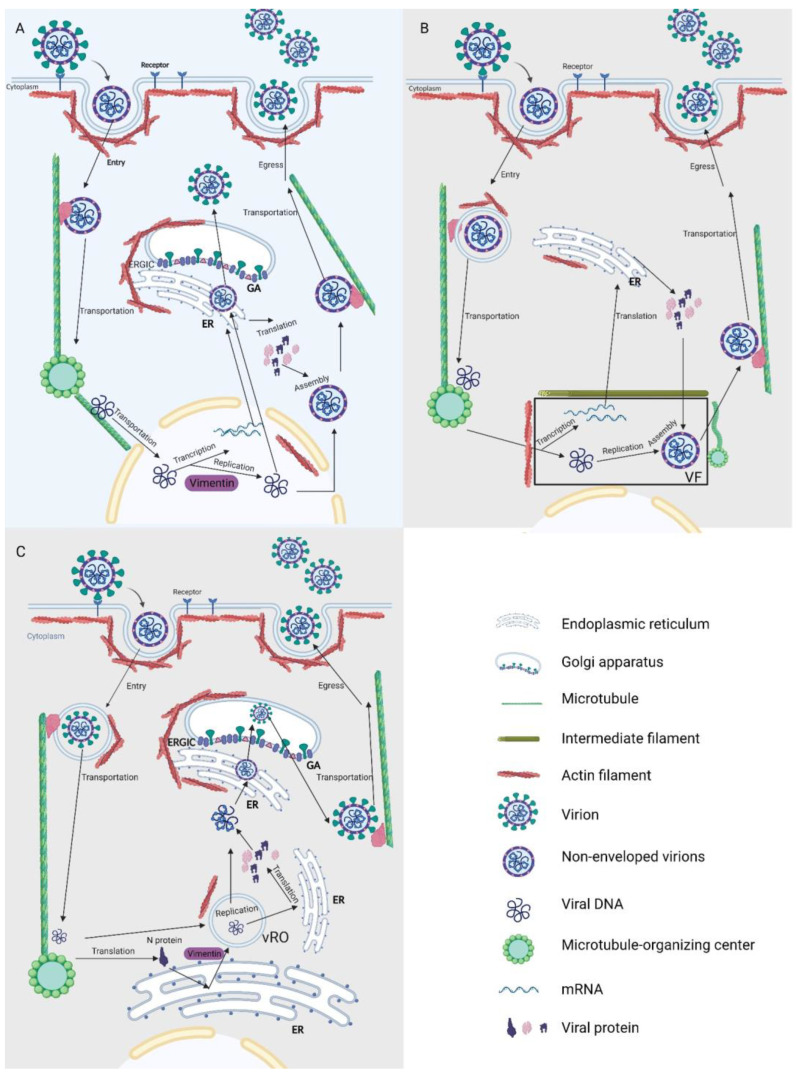
Schematic representation of different viruses using the cytoskeleton to complete their life cycle. (**A**) Similar to HSV, the process of cell entry requires actin rearrangement. After the viral genome enters the nucleus, replication and transcription begin to generate the nucleic acids and proteins required for viral assembly. This process is regulated by nuclear actin and vimentin. In the nucleus, viral proteins combine with DNA to form non-enveloped virions. Upon completion of assembly, virions either travel via ERGIC to produce mature virions or use microtubules for transport to the cell membrane and subsequent budding. (**B**) For poxviruses and African swine fever virus, the process of entry into cells requires actin rearrangement. The viral genome and related proteins are concentrated in the viral factory and begin to replicate and transcribe to generate the nucleic acids and proteins required for virus assembly. Actin/vimentin and microtubules are gathered around the viral factory. A large number of microtubule-organizing centers (MTOCs) are also present in the surrounding area. Subsequently, assembly is completed in the viral factory for microtubule-dependent transport out of the cell. (**C**) The cytoskeletal regulation of plus-strand RNA virus replication strategies is demonstrated. The process of entry into the cell requires actin rearrangement with subsequent transport to the ER region using microtubules. The viral genome is translated into the N protein, which is involved in the formation of vROs. Viral RNA replication is predominantly within vROs. Viral proteins assemble with RNA to generate non-enveloped virions, which undergo ERGIC to generate mature virions and are transported out of the cell in a microtubule-dependent manner. ER, Endoplasmic reticulum; GA, Golgi apparatus; ERGIC, ER/Golgi intermediate compartment structure; VF, virus factory; vROs, virus replication organelles.

AFs and MTs provide structural support for virus entry into the cell, the spatial configuration of endosomal membranes, intracellular transit, and recycling back to the cell surface, which are driven by different motor proteins. Vimentin regulates the transcription and translation of viral proteins [54]. In conclusion, the viral life cycle is greatly aided by alterations in the cytoskeleton and these effects vary depending on the virus species. The roles of cytoskeletal modifications are described in Figure 2 according to the stages of the viral life cycle.

### 4.1. Entry and Internalization

Viruses can enter cells via a variety of pathways, including membrane fusion and endocytosis. Viruses are usually captured by the pseudopodia of the cell, bind to their receptors, and enter the cell via membrane fusion [61]. In a recent report on SARS-CoV-2, cortical actin accumulation was observed in the plasma membrane of infected cells, suggesting the role of actin in virion entry, release, and transmission [62].

Actin and its regulators play an equally important role in endocytosis. When the human respiratory syncytial virus (RSV) and herpes simplex virus type 1 (HSV-1) infect, the viral capsid is surrounded by F-actin in synaptosomes, and actin is transiently depolymerized to form vesicles [63,64]. Many viruses enter cells via endocytosis with the help of clathrin, which requires actin for energy. The main mechanism by which RABV particles enter cells is clathrin-dependent [65], and viral particles enter cell inputs with elongated structures and an incomplete clathrin coating, which are dependent on actin for internalization [66]. The entry of virion-containing pits is hindered by actin disruption after pharmaceutical pretreatment with an actin-depolymerizing agent, such as latrunculin B or cytochalasin D, which does not prevent coated pit formation. The experimental phenomena of impeded infection demonstrate that the completion of the viral invasion process cannot be supported by clathrin on its own without actin to provide support [66]. Upon the arrival of the virus in the cell body, clathrin recruitment is initiated, and viruses undergo actin-mediated cell surfing to entry-specific sites. Notably, viral surfing continues during clathrin recruitment in pH-dependent viruses such as vesicular stomatitis virus (VSV) [67]. Surfing occurs along filopodia and AFs as they move toward endocytic hot spots. The movement of AFs in cell-surface protrusions, also known as F-actin reverse flow, involves myosin motors [68]. Myosin II is present in cellular processes that promote viral movement, which may affect the retrograde flow of F-actin from the filament group. Myosin II is the major ATPase involved in viral cell surfing [69].

In addition to the precise control of endocytosis by cortical actin, cell signal transduction is involved in the remodeling of the cytoskeleton after binding to the virus. During viral infection, Rho-GTPase signaling plays an important role in entry [70]; it is involved in regulating the actin structure, cytoskeleton assembly and remodeling, and mediating the phagocytosis of phagocytes with nucleating/elongation factors [71]. The glycoproteins of RSV and human parainfluenza virus type 3 (PIV-3) interact with RhoA to mediate viral entry [72]. Rac1 and Cdc42 are members of the RhoA-GTPase family involved in HSV-1 entry into neuronal and non-neuronal cells [73]. Another major signaling pathway is the phosphatidylinositol 3-kinase (PI3K)/protein kinase B (AKT), which plays a crucial role in cytoskeletal rearrangement. Dengue virus type 2 (DENV-2) infection induces AKT phosphorylation, leading to Rho activation and actin reorganization in Huh7 cells. The PI3K/AKT pathway is involved in DENV-2 infection in a Rho-GTPase and actin-dependent manner, and DENV-2 uses this signaling cascade to efficiently replicate in cells [74]. The third category is that most recently reported, showing that a disturbed actin cytoskeleton initiates the activation of pattern recognition receptors (PRRs). These sensor proteins are found in the cell membrane, nucleus, and cytoplasm. Many PRRs recognize certain viral or host-derived nucleic acids and, upon detection, cause the transcriptional activation of cytokines such as type I IFNs. Furthermore, PRRs are associated with cytoskeletal conversion. For instance, the retinoic acid-inducible gene I (RIG-I)-like receptor (RLR) pathway is activated following viral entry via actin rearrangement, a mechanism frequently linked to innate immunity [13].

No direct evidence has been reported regarding the association of IFs with viral invasion and transportation. Recent research has demonstrated that the establishment of human cytomegalovirus (CMV) infection is contingent upon the presence of an intact vimentin network, and that the cell tropism of CMV is contingent upon the integrity of the vimentin cytoskeleton [75].

### 4.2. Transport

MTs, AFs, and motor proteins are essential for generating the mechanical forces that drive the deformation and scission of cellular membranes. This mechanical activity facilitates the sorting of endosomal cargo and the generation of transport intermediates, enabling efficient intracellular transport processes. Virus transport by AFs and MTs often occurs shortly after entry into the cell membrane when it needs to traverse the inner layer of the cell membrane composed of microfilaments to inject its nucleic acid into the cell, or when the virus has completed replication and needs to be released from the cell membrane. Viral microfilament transport is a unidirectional movement that occurs after microfilament polymerization [76]. It has been demonstrated that the capsid protein and ICP0 of HSV-1 can interact with host MTs. Tubulin complex EB1 mediates the interaction between the viral capsid and the positive end of the microtubule, allowing the virus to undergo retrograde transport along the MTs upon entry [77]. ICP0 is a viral E3 ligase that destabilizes and unbundles MTs in Vero cells to aid in viral assembly and egress [78]. The destruction of AFs may hinder the assembly and egress of infectious bronchitis virus (IBV) [79]. Viruses can use MT trajectories as pathways for virions or as essential materials for virus assembly [80]. Viral proteins mediate the directional movement of virions along the MTs, which is important for viral transport out of the cell [81].

In fact, there are a few examples of viruses directly using MTs, more often using dynein and kinesin to transport viruses and cargo. Dynein interacts with the HSV-1 pUL37, and the key to its binding is the presence of a proline-rich domain in pUL37, and the interaction allows microtubules to smoothly transport the viral capsid [82]. The pUS9 of HSV-1 appears to use five arginine residues in its domain to bind to the host motor Kinesin-1 and contributes to anterograde axonal transport [83].

Interestingly, virions not only use motors for transport, but also dynein and kinesin on MTs to promote early infection [84]. The mature capsid (CA) core of human immunodeficiency virus 1 (HIV-1) encapsulates the viral genomic RNA, enzymes, and other viral proteins. The dynein motor complex is not only involved in the intracellular transport of the CA core, but may also be involved in the uncoating process of the CA core. After the downregulation of dynein expression, HIV-1 cDNA levels decrease, confirming that the HIV-1 reverse transcription is affected. However, the blockage of the CA take-off process by the virus caused by the downregulation of dynein expression appears to be transient, suggesting that uncoating is delayed rather than completely impaired [85]. Another study showed that multiple viral proteins directly interact with actin during HIV-1 infection, suggesting that HIV-1 may be anchored to cortical actin for reverse transcription and intracellular migration [86]. In conclusion, the retrograde transport of the HIV-1 CA core can effectively utilize dynein, while simultaneously using actin for transport.

### 4.3. Replication, Transcription, and Translation

During the replication of a positive-stranded RNA virus, viral replication organelles (vROs) composed of bilayer membrane-like structures are formed. The viral genomic RNA is wrapped in vROs to avoid the genome being degraded by other substances in the cytoplasm [87]. Live-cell imaging and sensors are used to monitor viral infections and replication, which shows that perinuclear inclusion in the SARS-CoV-2-infected cells is positive for dsRNA. Double-membrane vesicles (DMV), a form of vROs, are separated by reconstituted vimentin and encased in an IF cage. Drugs that inhibit vimentin also inhibit viral replication [88]. This cage is also surrounded by MTs that are excluded from the dsRNA-containing region, suggesting that IFs and MTs may serve to scaffold or confine the vROs compartment. Interestingly, bundles of cytoskeletal filaments have also been observed in the tomograms of infected cells in close proximity to vROs [62].

Notably, the formation of specialized replication organelles, or ‘viral factories’, has been observed in a range of viruses, including RNA viruses and large DNA viruses, such as poxvirus and African swine fever virus (ASFV). For these viruses, viral factories are typically organized around the periphery of the nucleus and serve as sites for efficient viral replication and assembly [83]. A study on vaccinia virus (VACV) has demonstrated that mRNA structures appear to be aligned on MTs, implying that MTs track connected mRNAs and cores. Accordingly, intact MTs are required for the typical punctate organization of viral mRNAs [89]. Early in infection, MTs retract toward the nucleus, rounding cell aggregates and bringing organelles close to the nucleus. Small early factories moved to the nuclear periphery in an MT-dependent manner to form larger factories [90].

Nuclear actin exists in the cell nucleus as a skeleton protein that participates in transcription, transcriptional regulation, and chromatin remodeling, and can control the nuclear expression of viral genes in the replication stages [91]. The movement of the HSV-1 capsid in the nucleus requires the participation of nuclear actin [92].

### 4.4. Assembly and Egress

VACV and ASFV assembly is consistent with viral replication and requires the involvement of a cytoskeleton-involved cytoskeleton-related perinuclear virus assembly factory [93]. Such cage structures, known as MTOCs, have been described above; their formation is important for the assembly of viral materials [94].

Newly synthesized viral proteins and nucleic acids in coronaviruses are transported to the actin-rich ER/Golgi intermediate compartment structure (ERGIC), which is adjacent to the endoplasmic reticulum and Golgi apparatus, facilitating the transfer of the virus from the endoplasmic reticulum to the Golgi apparatus, where the proteins undergo post-translational modification and complete the assembly stage [95]. Centrosomes are also important for the assembly of viruses; RNA viruses that replicate in the nucleus, such as retroviruses, can bud through centrosomes. For example, Foamy viruses must first accumulate in MTOCs and subsequently acquire an intact envelope via ERGIC to form intact virions [96].

Coronavirus-infected cells have been observed by electron microscopy, and actin parallel to the cell edge appears to be thickened [97]. The enhanced presence of actin can assist in providing a bending force to expel the progeny viral particles to the exterior [98]. Viral proteins are associated with the capsid proteins of viral particles, thus facilitating their ability to target sites of nuclear viral egress [99].

## 5. The Cytoskeleton Mediates Virus Transmission and Spread from Cell to Cell

**Figure 3:**
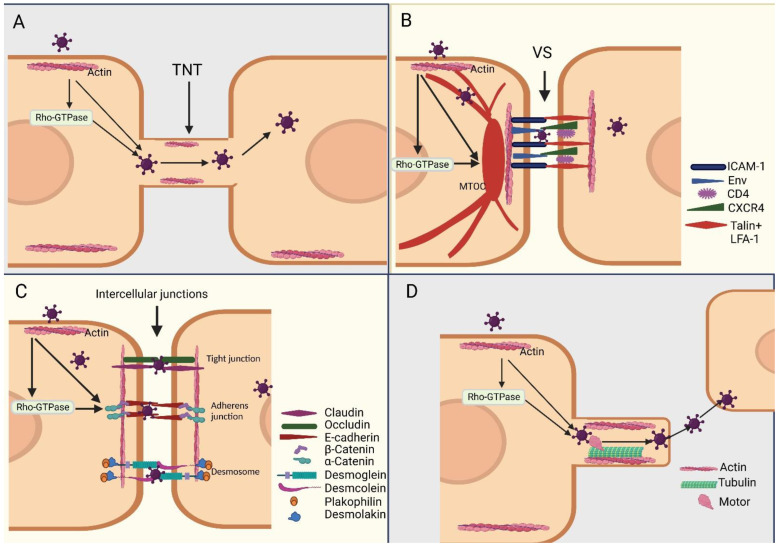
Schematic representation of the cell-to-cell spread of different viruses. (**A**) After viral infection, tunnel nanotubes are generated, and actin is required for this structure. The Rho receptor plays an important role during this period. TNT, tunnel nanotube. (**B**) During HIV infection, viral material is mobilized along microtubules to the site of cell–cell contact, where it traverses the central region of the synapse and enters the target cell. Upon arrival at the virological synapse (VS), LFA-1-talin complexes, and CD4 and CXCR4 are recruited in an actin-dependent manner. The VS is a specialized structure formed at the site of cell–cell contact, which facilitates the efficient transmission of HIV between infected and uninfected cells. VS, virological synapse. Env, HIV envelope protein; CXCR4, CXC chemokine receptor 4; ICAM-1, DC cognate ligands; LFA-1, lymphocyte function-associated antigen. (**C**) Intercellular junctions play a crucial role in maintaining the structural integrity and function of tissues. The three main types of intercellular junction are tight junctions, adherens junctions, and desmosomes. Tight junctions are formed by the transmembrane proteins claudin and occludin, which interact to form homotypic claudin–claudin and occludin–occludin complexes between adjacent cells. These transmembrane proteins bind directly to cytoplasmic adaptor proteins, which in turn associate with the actin cytoskeleton to maintain cell–cell adhesion and regulate the diffusion of ions and solutes through the tissue. Adherens junctions, on the other hand, are mediated by the transmembrane protein E-cadherin, which forms a ternary complex with the β-catenin and *α*-catenin. This complex binds to F-actin in a force-dependent manner, thereby regulating cell–cell adhesion and maintaining tissue integrity. Desmoglein and desmcolein bind to each other to form the core region. (**D**) Intracellular viruses can enter other cell-like comets due to slingshot-like structures formed by the intracellular microtubule and actin. Motor proteins are needed to empower this process.

Cell-to-cell transmission significantly boosts the effectiveness of viral transmission by concentrating the release of viral particles at the points of cell–cell contact [100]. It protects against antibodies that partially neutralize viruses [101], and under certain circumstances, overrides the inhibitory effects of specific antiviral restriction factors [102]. This viral transmission method may also affect the etiology and course of the infection [103]. It has been established that the cytoskeleton plays a major role in the transmission of viruses across cells (Figure 3).

### 5.1. Direct Transmission

The cells are linked by an open membrane channel called a tunnel nanotube (TNT) [104]. It can transmit a variety of items over great distances, including communication substances, genetic materials, and viruses. TNT can move not only small molecules, such as calcium ions, but also large molecules such as proteins, peptides, and organelles inside the cell [105]. With the help of this novel direct communication technique, the physiological and pathological aspects of various cell communication processes may be better understood, while also learning about novel long-distance communication mechanisms [105]. TNTs mostly consist of actin and MTs [106]. Despite ongoing research on TNT synthesis, the cytoskeleton plays a crucial role [104]. Moreover, Rho-GTPases play crucial signaling roles in this process [107]. It has been demonstrated that coronavirus, influenza virus, and HIV-1 cause TNT to develop and be transmitted between cells [107,108].

Virological synapse (VS) is a specialized site for the formation of virus-infected immune cells in contact with each other, and is a channel for the formation of contact between cells. The VS formation involves F-actin polymerization, depolymerization, and Rho-GTPase signaling [109]. Virions can be secreted from one cell to another via junctions [110]. After cell–cell contact, the cytoskeleton of infected cells rapidly polarizes to cell–cell junctions to form special sites at which different proteins are linked for virus transmission [111]. SARS-CoV-2 can spread between dendritic cells and target cells and invade nerve cells via connections similar to VS [112]. HIV-1 cell-to-cell transmission substantially increases the efficiency of viral transmission by concentrating the release of viral particles at the site of cell–cell interactions [113]. HIV-1 envelope proteins, such as Gag on the infected donor cells and CD4 on the uninfected target cells, interact to form VS, which requires actin support [114]. It has been suggested that during HIV infection viral particles are transported by MTs to cell-cell contacts, where they pass though the core region of the synapse and enter the target cell [110].

The structural continuity of tissues is maintained by three distinct types of cell–cell junction: desmosomes, tight and adherens junctions [115]. These junctions provide both extracellular and intracellular connections between neighboring cells, linking different elements of the cytoskeleton to form a cohesive structural network. In addition to their structural roles, these junctions are involved in regulating tissue integrity and controlling the diffusion of ions, solutes, and microorganisms through tissues [115]. It has been shown that hepatitis C virus and retroviruses may enter via tight junctions, human papilloma virus (HPV) may enter via adherens junctions, and HIV may modify gap junctions for entry [116].

### 5.2. Indirect Transmission

Many viruses use comets formed from actin to advance cytoplasmic viruses to the cell periphery or outside the infected cells [117]. Viral proteins may use actin-formed comet propulsion to pass through actin-enhanced cell junctions and be transported to neighboring cells [117]. The comet structure is essentially a slingshot structure formed by actin, which uses the elastic force of actin to push the virus out of the cell and facilitate its spread.

## 6. The Cytoskeleton Is Involved in the Immune Responses to Viral Infections

The cytoskeleton is also involved in innate immunity. Viral DNA is recognized by cyclic GMP-AMP synthetase (cGAS) [118], and viral RNA is recognized by retinoic acid- RLRs [119]. The recruitment of downstream molecules such as STING and MAVS results in the activation of downstream pathways. It can control gene expression, and its outcome is correlated with IFN production and expression [118,119].

The phosphorylation of RIG-I at Ser 8 and MDA5 at Ser 88 prevents RLR activation [120]. Once this site is dephosphorylated, RLRs are activated by the RNA viruses. The dephosphorylation of these sites by cellular protein phosphatase-1 PP1*α* or PP1*γ* is critical for RLR activation in response to viral infection [121], and virus-mediated perturbations of the actin cytoskeleton have been extensively documented to trigger RLR dephosphorylation via the PP1–R12C phosphatase complex [13]. Spire homolog 1 (Spir-1, also known as SPIRE1) has actin-binding domains, through which it nucleates actin filaments [122]. It has been demonstrated that Spir-1 stimulates innate immune signaling upon dsRNA sensing. Through a diphenylalanine motif, Spir-1 specifically contributes to the activation of interferon regulatory factor 3 (IRF3) and is also required for direct contact between Spir-1 and the VACV virulence factor K7. Spir-1 has been demonstrated to reduce VACV and ZIKV replication and/or dissemination, and is thus a virus restriction factor [123].

One example of a guanine nucleotide exchange factor (GEF) specific to RhoA, known as GEF-H1, is localized and confined to the MTs. This sequestration is associated with the precise temporal and spatial activation of Rho-GTPases [124]. Inactive GEF-H1 binds to the dynein motor complex on MTs, and GEF-H1 is activated and released from MTs upon cellular interactions, contributing to the recognition of intracellular pathogens. GEF-H1 can function in the RLR pathway in conjunction with MAVS and TANK-binding kinase 1 (TBK1); the inhibitor of nuclear factor-kappa B (I*κ*B) kinase epsilon (IKK*ε*) complexes to enhance the phosphorylation of IRF3 and the activation of the *ifnb1* promoter [125].

**Figure 4:**
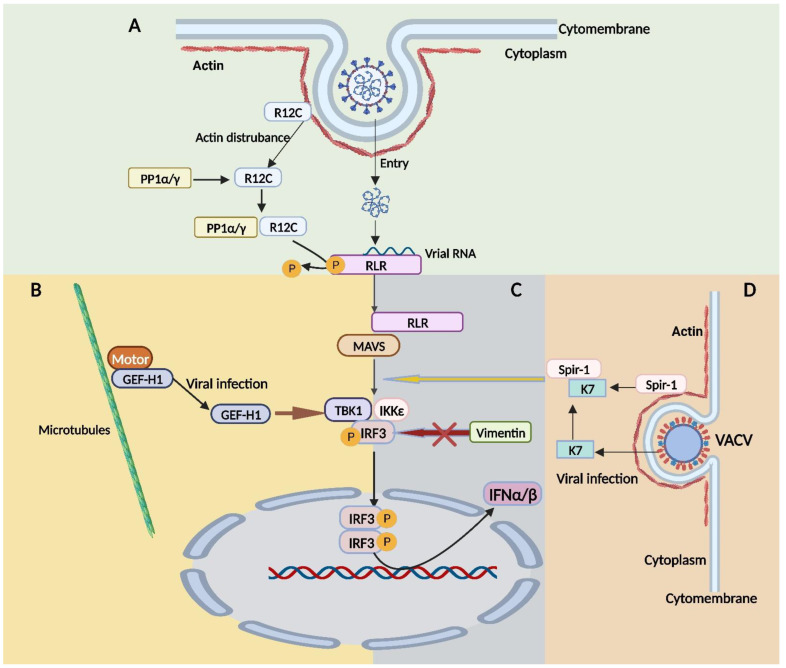
Involvement of the cytoskeleton in the immune response to viral infections. The inclusion of actin, the microtubule, and vimentin affects the regulation of the RLR signaling pathway in interferon production. (**A**) R12C can be released from the actin phosphatase complex during viral infection and participate in the binding of RLRs to PP1*α*/*γ*. (**B**) GEF-H1 can be released from the microtubule motor protein complex during viral infection and affect the phosphorylation of IRF3. (**C**) Vimentin can regulate TBK1 and IKK*ε*, and affect the phosphorylation and nuclear import of IRF3. (**D**) The actin nucleating factor Spir-1 interacts with the K7 protein of vaccinia virus upon infection, promoting the IRF3 activation downstream of MAVS and upstream of IKK or TBK1. RLRs, retinoic acid-inducible gene I (RIG-I)-like receptor. R12C, a regulatory subunit of the protein phosphatase 1 (PP1). GEF-H1, guanine nucleotide exchange factor. IRF3, interferon regulatory factor. TBK1, TANK-binding kinase 1. IKK*ε*, I*κ*B kinase epsilon.

Recently, vimentin has been reported to play a role in many vital immune responses processes, and it has been described as a ligand for some PRRs. Vimentin expression may depend on IFNs [14], and viral infection may promote vimentin promoter activity. Vimentin overexpression is accompanied by enhanced viral replication, and the inhibition of IRF3 and TBK1 phosphorylation. Vimentin has been suggested to suppress the production of type I IFNs by targeting IRF3 or its associated binding partners, including TBK1 and inhibitors of I*κ*B kinase epsilon (IKK*ε*) [17]. During a viral infection, TBK1, IKK, and IRF3 form a complex. Once activated, TBK1 and IKK phosphorylate IRF3 to enhance its nuclear translocation. Both TBK1 and IKK possess an N-terminal kinase domain (KD) or interaction with IRF3 [126]. Vimentin and IRF3 bind to the KD domain of TBK1 or IKK, which may prevent the formation of the TBK1–IKK–IRF3 complex and the nuclear translocation of IRF3 [15,126]. The above three examples of RLR signaling pathways being affected are summarized in Figure 4.

Nucleotide oligomerization domain-containing protein 2 (NOD2) is an important receptor involved in cellular innate immunity, and vimentin is an NOD2-interacting protein in mammalian cells. A recent study has suggested that NOD2 interaction with vimentin is important for its ability to respond to the signals downstream of NF-*κ*B [127].

## 7. Conclusions and Prospects

In this review, we provide a comprehensive overview of the structure and biological functions of the cytoskeleton, focusing on changes in the cytoskeleton during viral infection and whether these changes affect the virus life cycle. Organisms are complex and the interactions between the cytoskeleton and viruses are ingenious. The cytoskeleton also resists the processes of invasion and replication through the cytoskeleton-mediated innate immune response upon viral infection.

For example, after infection with certain RNA viruses, actin is rearranged and R12C is released to regulate RLRs. This reveals that the cytoskeleton is like a warehouse containing a large number of signal regulatory substances. Once stimulated, a large number of enzymes and proteins are released during the process of depolymerization. Some of these substances can be used by cells to generate innate immunity to initiate signaling processes. Currently, there are no data to confirm whether other PRRs, such as cGAS-STING, have similar upstream signaling regulators. Obtaining these data is recommended for the study of broad-spectrum antiviral drugs.

In addition, we showed that the virus uses motor proteins to transport virions to the correct replication site during the invasion process, and the example of GEF-H1 demonstrates that the binding of motor proteins to the virus simultaneously engages GEF-H1 in the antiviral response. It is unclear whether the cytoskeleton regulates both the viral life cycle and the innate immune processes. Currently, no exact data are available, and this is worthy of further investigation.

Viral infection causes cytoskeleton alterations that can be either particular or general. During viral invasion, the membrane deforms and actin rearranges. R12C, which specifically binds to RLRs, can be released by actin disturbance via a non-specific pathway [13]. Vimentin, for instance, can participate in the invasion of viruses as a co-receptor (specific) [128] and impede the entry of HPV16-PsV, demonstrating that it can prevent virus–receptor contact through steric hindrance (non-specific) [129]. Additionally, upon viral infection, vimentin expression is increased, which may be influenced by IFN receptor 1 (IFNAR1), a nonspecific factor triggered by almost all viral infections, can inhibit TBK1 and IKK*ε* during the process of enhanced vimentin expression [15]. Hence, the differences between non-specific and specific cytoskeleton-mediated activation need to be clarified.

In conclusion, the cytoskeleton is involved in almost all physiological processes in the cell, and therefore its roles in the process of virus invasion are inevitable. In the future, we will continue to study the cytoskeleton to provide insights into the design and development of antivirals.

## Figures and Tables

**Table 2:** Pathological roles of the cytoskeleton.

Types of Pathogeneses	Changes in the Cytoskeleton	The Effects of the Changes	Pathological Roles	References
Cancers	Depolymerization and polymerization of actin	Contributing to cell migration	Devoting to cancer cells spread and replicate quickly	[41,42,43,44,45]
	Depolymerization, polymerization and modification of microtubules	Participating in cell movement through signal transduction and as a transport structure		
	Interaction of vimentin with actin and microtubules.	Contributing to cell–matrix adhesion and migration		
	Activation of vimentin expression, and interaction of vimentin with motor proteins	Aims to enhance cell motility, which is conducive to the process of epithelial–mesenchymal transition (EMT)		
Intracellular bacteria infected	Actin is recruited and interacts with actin regulatory factors Arp2/3	Leading to bacterial engulfment and internalization in a membrane-bound vacuole	Promoting the infection of intracellular bacteria	[46,47,48,49,50,51,55]
	Microtubule depolymerization and the activity of the Rho family of enzymes that control microtubules are affected and interfered with by bacterial production of *Clostridium difficile* toxin A (TcdA)	Participating in bacterial transportation and the consequential immune-inflammatory responses		
	Vimentin is expressed on the cell surface, secreted and located extracellularly	Contributing to stress reaction; vimentin can be both pro- and anti-bacterial, favoring bacterial invasion in some contexts, but also involved in bacterial-induced inflammation regulation		
Viruses infected	Actin depolymerizes and polymerizes, and kinetoproteins are recruited.	Contributing to entry and internalization	Assisting the virus to complete its life cycle	[15,52,53]
	Microtubule and motor proteins interact with viral proteins, microtubule depolymerization and polymerization, motor proteins are changed	Transporting viral components, formation of replicative organelles		
	The vimentin expression is changed	Contributing to viral replication and signaling		
Parasites infected	Plasmodium can promote actin polymerization in vitro	Inhibiting the movement of cargo vesicles to the erythrocyte plasma membrane	Promoting severe *Plasmodium falciparum* malaria infection	[54]

## Data Availability

All data generated or analyzed during this study are included in this published article.
